# Intra-session and inter-rater reliability of spatial frequency analysis methods in skeletal muscle

**DOI:** 10.1371/journal.pone.0235924

**Published:** 2020-07-10

**Authors:** Scott K. Crawford, Kenneth S. Lee, Greg R. Bashford, Bryan C. Heiderscheit

**Affiliations:** 1 Department of Orthopedics & Rehabilitation, University of Wisconsin-Madison, Madison, Wisconsin, United States of America; 2 Department of Radiology, University of Wisconsin-Madison, Madison, Wisconsin, United States of America; 3 Department of Biological Systems Engineering, University of Nebraska, Lincoln, Nebraska, United States of America; University of Montreal, CANADA

## Abstract

Spatial frequency analysis (SFA) is a quantitative ultrasound (US) method originally developed to assess intratendinous tissue structure. This method may also be advantageous in assessing other musculoskeletal tissues. Although SFA has been shown to be a reliable assessment strategy in tendon tissue, its reliability in muscle has not been investigated. The purpose of this study was to examine the reliability of spatial frequency parameter measurement for a large muscle group within a healthy population. Ten participants with no history of lower extremity surgery or hamstring strain injury volunteered. Longitudinal B-mode images were collected in three different locations across the hamstring muscles. Following a short rest, the entire imaging procedure was repeated. B-mode images were processed by manually drawing a region of interest (ROI) about the entire muscle thickness. Four spatial frequency parameters of interest were extracted from the image ROIs. Intra- and inter-rater reliabilities of extracted SFA parameters were performed. Test-retest reliability of the image acquisition procedure was assessed between repeat trials. Intraclass correlation coefficients showed high intra- and inter-rater reliability (ICC(3,1) > 0.9 for all parameters) and good to moderate test-retest reliability (ICC(3,1) > 0.50) between trials. No differences in parameter values were observed between trials across all muscles and locations (p > 0.05). The high reliability metrics suggest that SFA will be useful for future studies assessing muscle tissue structure, and may have value in assessing muscular adaptations following injury and during recovery.

## Introduction

Imaging modalities such as ultrasound (US) are typically used in conjunction with non-imaging clinical tests to aid in diagnoses of various musculoskeletal (MSK) conditions [1,2]. Measures of quantitative US, such as echo-intensity or first-order statistical analysis of individual image pixels [3–7], have been correlated to muscle quality and function in both animal models and humans [6,8–11].

Ultrasound is a particularly useful imaging modality because it can reveal tissue organization at a greater resolution than other modalities [12,13]. For example, the same speckle pattern that limits apparent spatial resolution in US can indirectly show tissue health in tendon [14–16]. Spatial frequency analysis (SFA) is a quantitative US method, which leverages the coherent imaging properties of US by analyzing the cumulative reflected image pattern. The prominent spatial frequencies within an image show the overall organization of the underlying tissue hierarchy. These spatial frequency characteristics have successfully differentiated pathological and healthy tendons and correlated collagen organization to tendon properties *in vivo* [14,16]. Therefore, SFA builds upon other grayscale analysis methods by characterizing the two-dimensional speckle pattern of MSK tissue images such as tendon and muscle, rather than just analyzing individual pixels.

To date, SFA has only been applied to tendons. It is of interest to determine if SFA methods may be reliably used in other MSK tissues such as muscle. By measuring the reliability of SFA for muscle, future studies investigating differences in muscle architecture due to injury or during rehabilitation may be conducted with more confidence. The objectives of this study were to examine the use of SFA for assessment of muscle tissue, specifically by 1) determining the intra- and inter-rater reliability of extracted SFA parameters and 2) determining the test-retest reliability of SFA parameters within the hamstring muscles in a healthy population.

## Materials and methods

An *a priori* power analysis was performed using R software [17] based upon the methodology proposed by Zou [18] using a hypothesized value of 0.75, a null hypothesis value of 0, alpha of 0.05, power of 0.80, and number of ratings of each subject of 2. A total sample size of N = 10 subjects was determined. Ten participants from the university community were recruited to participate in this study. Inclusion criteria were: 18–35 years of age, regularly participating (three or more days per week) in exercise or recreational sport; no history of lower extremity surgery or hamstring strain injury; and not being currently pregnant. This study was approved by the Health Sciences Institutional Review Board at the University of Wisconsin-Madison and all participants provided written informed consent.

### Ultrasound imaging

Participants were positioned prone on an exam table with their hips and knees supported in neutral position and then asked to remain relaxed with no muscular contraction. In an effort to standardize imaging locations between participants, thigh length from the ischial tuberosity to the midpoint between the femoral condyles was measured and recorded. Skin marks were then made on the participant at 33%, 50%, and 67% of the thigh length from the ischial tuberosity, which corresponded to approximately proximal, mid-belly, and distal regions of the hamstring muscle, respectively. These locations were determined based upon pilot testing to ensure that images were collected at these different regions with minimal tendon infiltration, and are consistent with previous investigations [19–22].

All images were obtained using the same machine (Aixplorer, Supersonic Imaging, Weston, FL) and sonographer with over 18 years of US experience (5+ years in MSK US). A linear array transducer (2–10 MHz) was used with the following parameters: imaging depth of 5 cm, dual transmit foci depth of 2 and 3 cm (corresponding to approximately the center of the muscle [23]), and gain of 38%, as this was determined from preliminary image acquisitions to result in clear images without image saturation. All ultrasound settings were kept constant for all image acquisitions [6,24].

Ultrasound gel was liberally applied at each imaging site. To ensure that the targeted HS muscle was imaged, a transverse view was first visualized after ensuring the ultrasound probe was placed at the appropriate location with respect to the ischial tuberosity. Longitudinal B-mode images were then captured for each hamstring muscle (biceps femoris long head, BFlh; semitendinosus, ST; semimembranosus, SM) at each of the three locations along both thighs. Biceps femoris short head (BFsh) was excluded from the analysis primarily because BFsh is deep to BFlh, thereby making imaging difficult [25]. After image acquisitions were completed at all locations from both limbs, the participant sat on the exam table for 60 seconds, before laying back down. The same imaging procedure was then repeated for each limb.

### Image analysis

The static B-mode images were saved on the local computer and extracted for subsequent analysis. Longitudinal images were processed using custom MATLAB algorithms (Mathworks, Natick, MA). For all image analyses, a polygonal region of interest (ROI) was drawn about the central portion of the muscle of interest with the superficial and deep boundaries of the ROI drawn approximately 3–4 mm from the aponeuroses [26].

**Fig 1:**
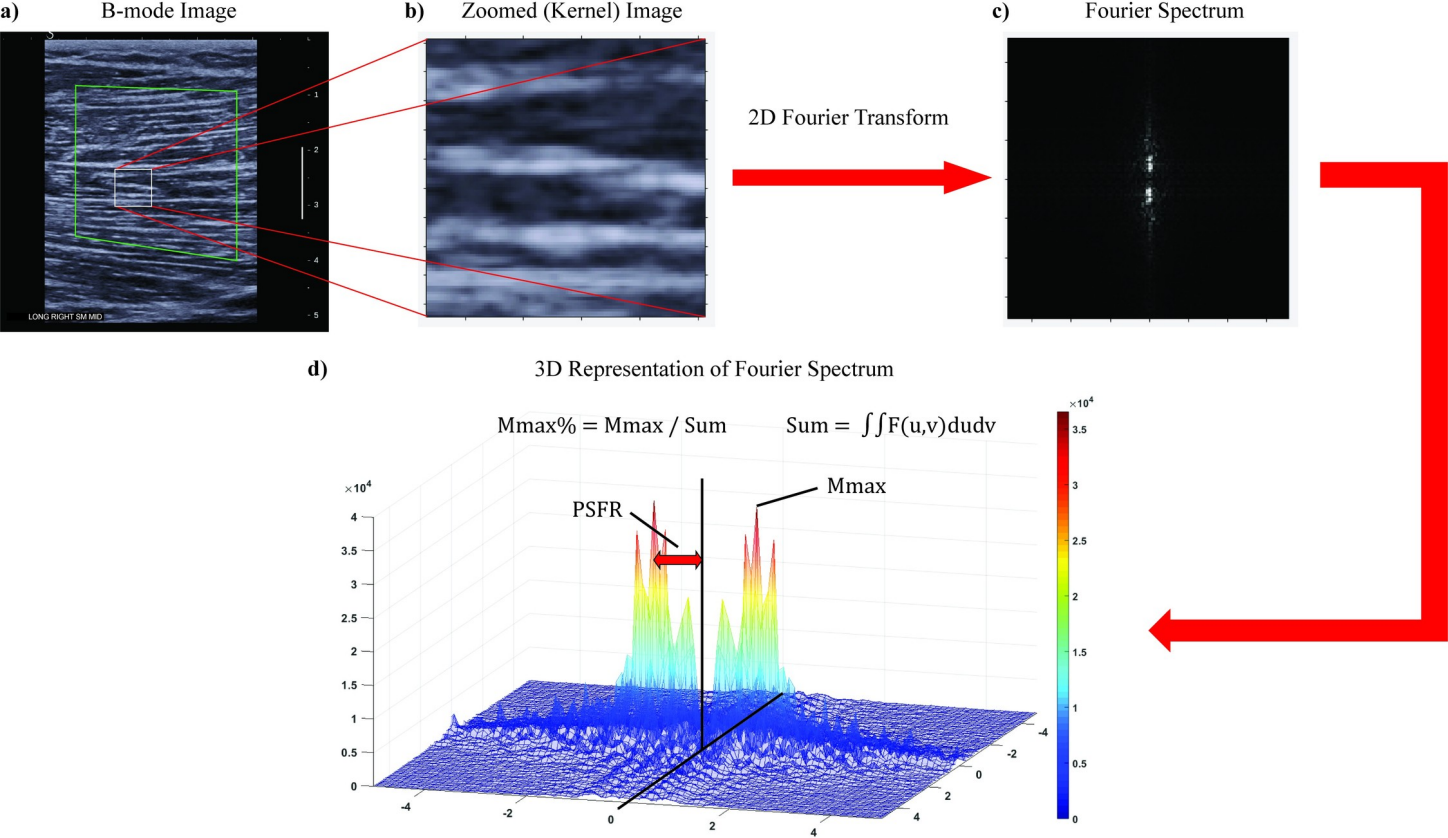
SFA procedure. a) Representative B-mode image of the mid-belly of the semimembranosus with ROI and kernel. The larger green polygon is a representation of the segmented parent ROI. The ROI was drawn in the central portion of the muscle by identifying the superficial and deep (when visible) aponeuroses being careful to avoid boundary effects of the aponeuroses and lateral edges of the image. The smaller white square is an example of a kernel in which the 2D Fourier Transform was performed. b) Zoomed in image of kernel within B-mode image. c) 2D Fourier spectrum of kernel. d) 3D representation of Fourier spectrum with SFA parameters visualized.

All possible 96 x 96 pixel sub-images (“kernels”) within the ROI, which correspond to a square with 6.6 mm sides, were analyzed in the spatial frequency domain. The kernel size was determined from pilot data of previously collected hamstring images by observing the cumulative image pattern of several fascicles within the kernel. A 2D Fourier Transform was applied to each kernel after zero-padding to 128 x 128 samples to increase frequency sampling. A 2D highpass filter (-3 dB cut-off about 1.0 mm^-1^) was then applied to attenuate low spatial frequency artifacts. The kernels were permitted to overlap and the spatial frequency parameters [14,27] were extracted and averaged over all kernels of the ROI. Thus, spatial frequencies were analyzed in both the axial and lateral directions and a single value was obtained for each parameter for the entire ROI (Table 1, Fig 1).

**Table 1:** Mathematical formulations, descriptions, and physiological correlate of spatial frequency parameters. Adapted from Pearson et al. (2017).

Parameter	Mathematical Formulation	Mathematical Description	Tissue Indicator(s)
**Peak spatial frequency radius (PSFR)**	$\sqrt{u_{max}^{2}+v_{max}^{2}}$, where u_max_, v_max_ are directional vectors in frequency domain	Distance from origin to peak of maximum frequency amplitude in 2-D Fourier spectrum	Dominant frequency of banded fascicular organization pattern
**Mmax**	*max*(*F*(*u,v*))	Value of the maximum frequency amplitude in 2-D Fourier spectrum	Strength of frequency of most prominent banded pattern of tissue
**Mmax Percent**	max(F(u,v))∬F(u,v)dudv	Ratio of Mmax to total intensity of pixels in 2-D Fourier spectrum	Contribution of most prominent fascicle pattern compared to total background
**Sum**	∬*F*(*u,v*)*dudv*	Sum of frequency amplitudes in 2-D Fourier spectrum	Equivalent to total image brightness

### Reliability procedure

**Fig 2:**
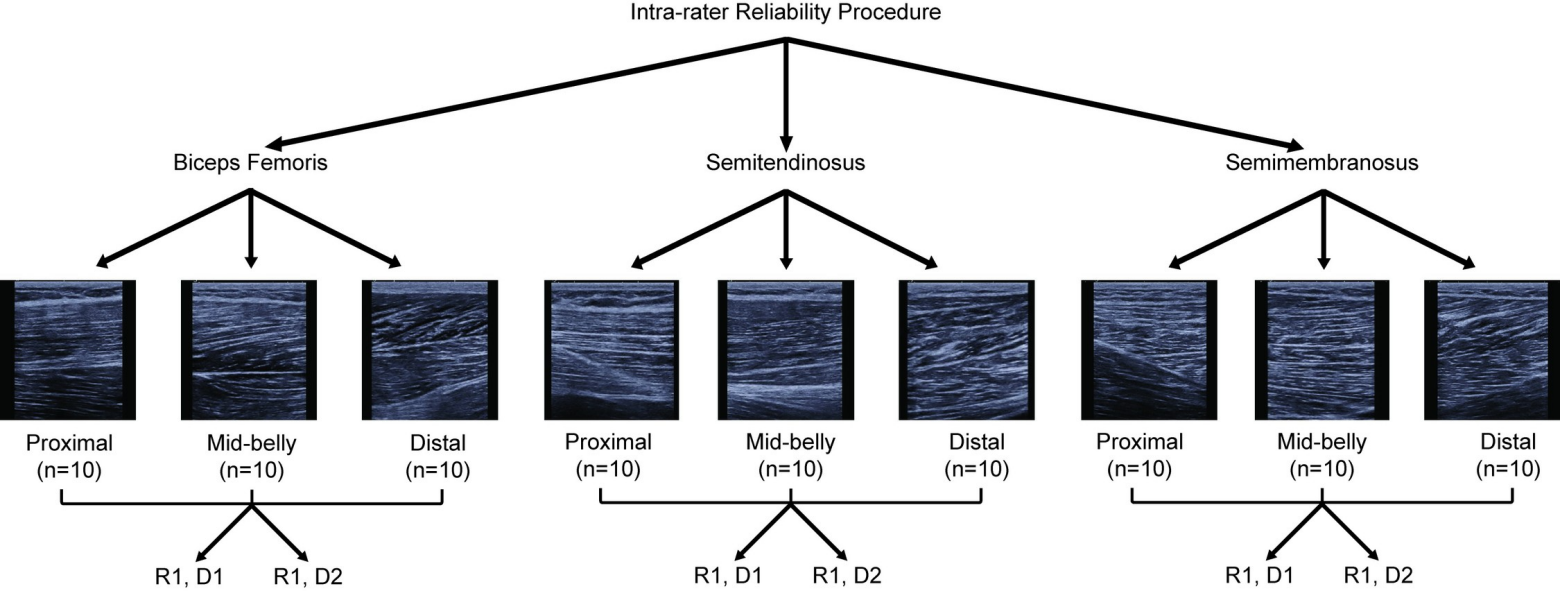
Flow chart of intra-rater image analysis reliability protocol. The same rater performed the ROI segmentation and SFA parameter extraction on the same images on two different days to determine the intra-rater reliability. D1 = day 1, D2 = day 2, R1 = rater 1.

**Fig 3:**
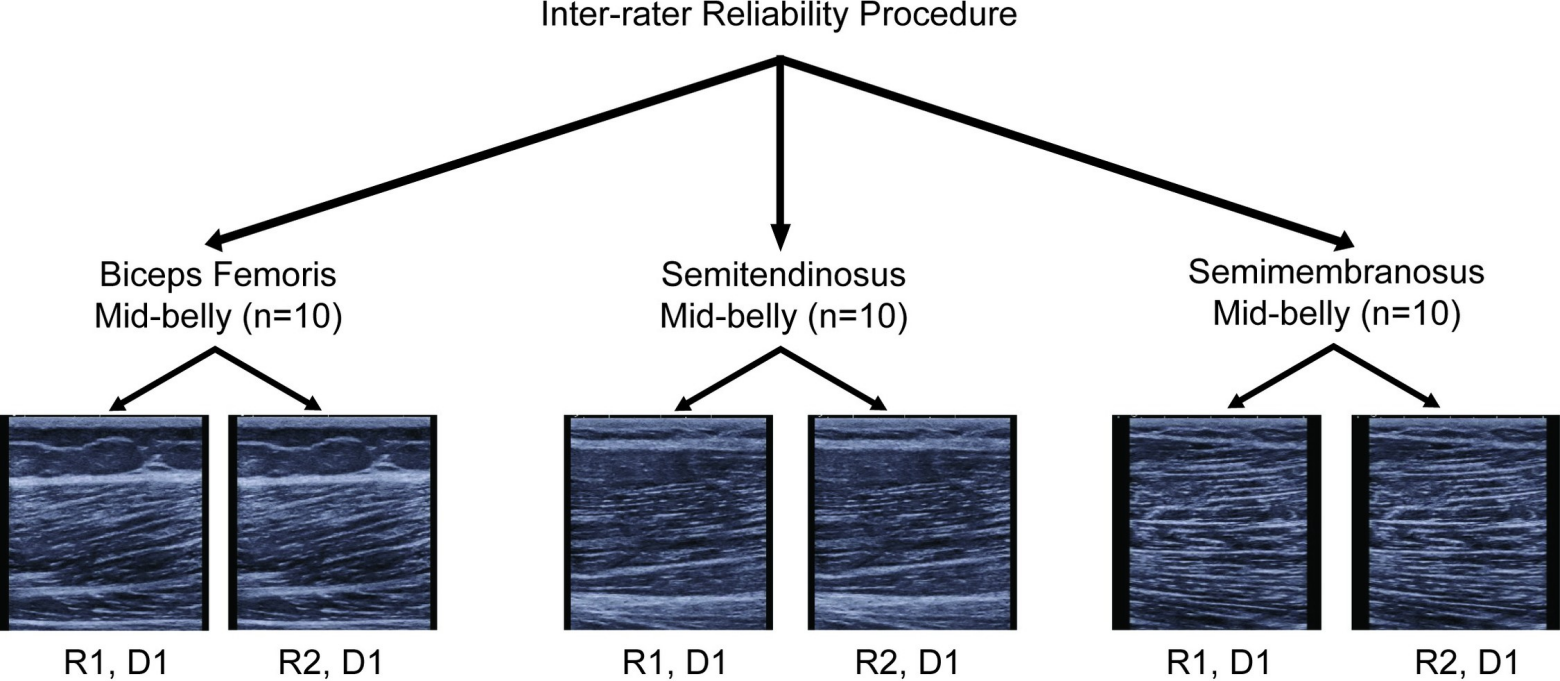
Flow chart of inter-rater image analysis reliability protocol. Two different raters performed the ROI segmentation on the same images and spatial frequency parameters were extracted and compared between each rater’s ROI. D1 = day 1, R1 = rater 1, R2 = rater 2.

**Fig 4:**
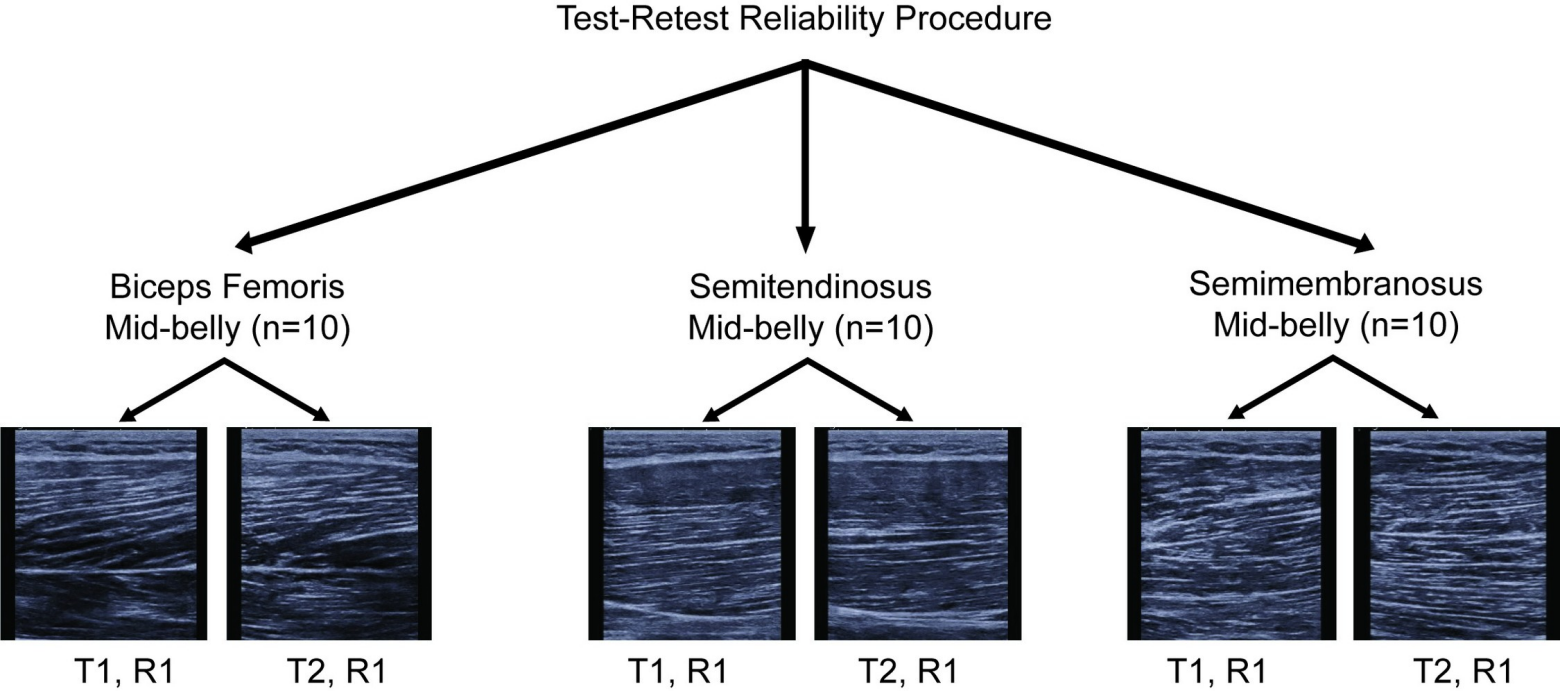
Flow chart of test-retest image analysis reliability protocol. Images from the repeat image acquisition at the mid-belly were analyzed by the same rater. R1 = rater 1, T1 = trial 1, T2 = trial 2.

The reliability assessment protocol was divided into three parts. First, the intra-rater reliability was performed to assess the reliability of extracting spatial frequency parameters from ROIs drawn on the same images on two different days by the same rater (S.K.C.) (Fig 2) [26]. A total of 10 images per muscle and location combination were randomly selected for this analysis (90 images total). Second, the inter-rater reliability was performed to compare the extracted spatial frequency parameters from ROIs drawn by two different raters on the same image (Fig 3). A total of 30 images from the mid-belly location were randomly selected for analysis. The mid-belly location was chosen since most HS imaging studies assessing architectural measures are typically performed at the mid-belly of the muscle [19,28–30]. The use of this location is also consistent with previous investigations in architectural reliability measures [26]. Therefore, the SFA method implemented at the mid-belly was performed as a means of comparison to previously reported protocols. The raters were instructed to draw ROIs within the central region in each image. Third, the reliability of the entire image acquisition procedure (test-retest) and SFA method was assessed between the repeat trials at the mid-belly of each muscle from each limb of each participant resulting in 60 images analyzed (Fig 4).

### Statistical analysis

A two-way, mixed effects intraclass correlation (ICC) for absolute agreement (ICC(3,1)) was used to assess the intra-rater reliability of each spatial frequency parameter (Table 1) between the same images. To assess agreement in the ROI segmentation of the muscles between raters, the ROI was converted into a binary image where the enclosed ROI was white and the remainder of the image was black. The area of each polygonal ROI and a Sørensen-Dice similarity coefficient were calculated in MATLAB to compare the pixels of the converted binary images [31]. This was calculated as $dice(A,B)=\frac{2*|intersection(A,B)|}{\left|A|+|B\right|},$ where A and B are the set of pixels in the first and second images, respectively, and |A|, |B| are the cardinal sets of A and B, respectively. The area of the pixels contained within the ROIs of each rater was calculated and compared. Two-way random, single rater ICCs for consistency (ICC(2,1)) was performed to determine the reliability between ROI areas and the extracted spatial frequency parameters from the ROIs drawn by different raters. A two-way, mixed effects ICC for absolute agreement (ICC(3,1)) was used to determine the test-retest reliability of the entire image acquisition protocol. Standard error measurement (SEM, SEM%) of each spatial frequency parameter was calculated as $SEM=\sqrt{{MS}_{E}}$, where MS_E_ was the mean square error term from the ANOVA table, as this has previously been suggested to allow for more consistent interpretation of SEM values across studies [32,33]. The SEM % was defined as SEM%=(SEMMean(Trial1,Trial2))x100% [34]. Paired t-tests were performed to compare parameter values between trials. The level of reliability was defined as poor (ICC < 0.5), moderate (0.5 < ICC < 0.75), good (0.75 < ICC < 0.9), or excellent (ICC > 0.9) for both intra-session and inter-rater reliability measures [35,36]. All analyses were performed in IBM SPSS Statistics for Windows, Version 25.0 (IBM Corporation, Armonk, NY).

## Results

The ten participants (6 males, 4 females) had a mean ± standard deviation (SD) age of 23.4 ± 1.2 years, body mass 69.9 ± 9.3 kg, height 174.2 ± 6.7 cm, and BMI 23.0 ± 3.0 kg/m^2^. The sizes of the ROIs drawn by the same rater on the same image were all within a mean 4.8 ± 7.8% absolute difference in the number of kernels across all muscles and locations. The intra-rater ICC reliability measures for the extracted spatial frequency parameters were excellent (ICC(3,1) > 0.90) for all muscle locations (Table 2).

**Table 2:** Intra-rater intraclass correlation coefficients (ICC(3,1) [95% CI]) by muscle and location.

Muscle	Location	PSFR	Mmax	Mmax %	Sum
**Biceps Femoris**	Proximal	0.997 [0.990, 0.999]	0.996 [0.985, 0.999]	0.998 [0.991, 0.999]	0.998 [0.991, 1.00]
	Mid-belly	0.998 [0.994, 1.00]	0.999 [0.995, 1.00]	1.00 [0.999, 1.00]	0.999 [0.995, 1.00]
	Distal	0.994 [0.976, 0.998]	0.998 [0.991, 0.999]	0.996 [0.985, 0.999]	0.991 [0.968, 0.998]
**Semitendinosus**	Proximal	0.996 [0.983, 0.999]	0.997 [0.990, 0.999]	0.994 [0.975, 0.999]	0.998 [0.990, 1.00]
	Mid-belly	0.997 [0.987, 0.999]	0.994 [0.976, 0.998]	0.993 [0.974, 0.998]	1.00 [0.999, 1.00]
	Distal	0.998 [0.987, 1.00]	0.999 [0.996, 1.00]	0.998 [0.991, 0.999]	0.999 [0.997, 1.00]
**Semimembranosus**	Proximal	0.995 [0.977, 0.999]	0.999 [0.998, 1.00]	1.00 [0.999, 1.00]	1.00 [0.995, 1.00]
	Mid-belly	0.980 [0.928, 0.995]	0.999 [0.996, 1.00]	1.00 [0.998, 1.00]	1.00 [0.999, 1.00]
	Distal	0.999 [0.995, 1.00]	0.995 [0.979, 0.999]	0.982 [0.935, 0.996]	0.997 [0.989, 0.999]

PSFR = peak spatial frequency radius

The mean absolute difference between the areas of each ROI of different raters was 16.8 ± 20%. Dice coefficients ranged from 0.53 to 0.98 with a mean ± SD of 0.90 ± 0.10. The agreement between ROI segmentation between different raters was good with an ICC(2,1) = 0.79 (95% CI = 0.57, 0.90). The reliability between raters of extracted spatial frequency parameters was excellent for PSFR (ICC(3,1) = 0.950 (95% CI = 0.894, 0.976)), Mmax (ICC(3,1) = 0.979 (95% CI = 0.955, 0.990)), Mmax % (ICC(3,1) = 0.984 (95% CI = 0.966, 0.992)), and Sum (ICC(3,1) = 0.977 (95% CI = 0.953, 0.989)).

The reliability of the entire procedure at the mid-belly was good to moderate (ICC(3,1) > 0.50) across all parameters and muscles with the exception of Mmax in BFlh (ICC(3,1) = 0.45) (Table 3). The SEM % for all parameters across all locations was less than 15% (Table 3, range 6.3 to 14.9%). Mean and SD values for each parameter for each trial are shown in Table 4. There were no differences in parameter values between trials across all muscles and locations (p = 0.06–0.95) (Table 4).

**Table 3:** Results of test-retest procedure by muscle at the mid-belly.

Muscle	Parameter	ICC(3,1) [95% CI]	SEM	SEM %
**Biceps Femoris**	*PSFR*	0.85 [0.62, 0.94]	0.05	6.3%
	*Mmax*	0.45 [0, 0.78]	1,776	14.9%
	*Mmax%*	0.55 [0, 0.82]	0.24	14.3%
	*Sum*	0.79 [0.48, 0.92]	58,005	8.0%
**Semitendinosus**	*PSFR*	0.84 [0.60, 0.97]	0.06	7.0%
	*Mmax*	0.92 [0.79, 0.97]	746	7.6%
	*Mmax%*	0.92 [0.80, 0.97]	0.13	8.2%
	*Sum*	0.81 [0.54, 0.93]	25,102	8.0%
**Semimembranosus**	*PSFR*	0.86 [0.65, 0.95]	0.05	6.9%
	*Mmax*	0.69 [0.24, 0.87]	1,303	11.9%
	*Mmax%*	0.90 [0.74, 0.96]	0.15	8.4%
	*Sum*	0.85 [0.64, 0.94]	51,242	8.1%

PSFR = peak spatial frequency radius

^a^SEM % was calculated as (SEMMean(Trial1,Trial2))x100%.

**Table 4:** Means and standard deviations by test-retest trial and spatial frequency parameter.

	PSFR			Mmax			Mmax %			Sum		
	*Trial 1*	*Trial 2*	*P-value*^*a*^	*Trial 1*	*Trial 2*	*P-value*	*Trial 1*	*Trial 2*	*P-value*	*Trial 1*	*Trial 2*	*P-value*
**Biceps Femoris**	0.76 (0.13)	0.73 (0.12)	0.17	11,537 (2,165)	12,376 (2,611)	0.20	1.65 (0.37)	1.70 (0.35)	0.61	713,632 (109,316)	744,377 (143,134)	0.21
**Semitendinosus**	0.87 (0.15)	0.87 (0.16)	0.83	9,599 (2,635)	10,078 (2,566)	0.14	1.52 (0.44)	1.56 (0.45)	0.49	642,976 (126,466)	660,056 (117, 008)	0.44
**Semimembranosus**	0.76 (0.15)	0.76 (0.15)	0.92	10,474 (2,207)	11,463 (2,393)	0.06	1.74 (0.41)	1.85 (0.53)	0.07	617,147 (130,768)	645,879 (138,721)	0.19

PSFR = peak spatial frequency radius

^a^ P-values were calculated from paired t-tests for each parameter

## Discussion

This study is the first to investigate the use of SFA for assessment of muscle tissue. The intra- and inter-rater reliability of the SFA image analysis method and subsequent parameter extraction were excellent. The test-retest procedure reliability was good to moderate.

The intra-rater reliability of the extracted spatial frequency parameters was nearly unity. Furthermore, the reliability was not dependent upon the muscle (BFlh, ST, or SM) or the location along the muscle length. This highlights the fact that when the same rater performs the SFA procedure on similar images, then it can be expected that similar results will be obtained. This is an important factor when adapting this method for assessment of muscle structure between different muscles and within different locations along the length of the same muscle. The high ICC values for the intra-rater reliability are consistent with some findings using first-order gray-scale statistics and backscatter analysis in healthy subjects [6,23,37,38] and the SFA method in Achilles tendons [39].

Similarly, the reliability of extracted spatial frequency parameters between raters was excellent and is consistent with other SFA investigations in the supraspinatus [40]. This is particularly noteworthy since the repeatability of the ROI segmentation between raters had less agreement compared to ROIs drawn by the same rater (ICC = 0.79 vs 0.98, respectively). Despite the difference in ROI segmentation between raters (difference between raters was approximately 17%), similar spatial frequency parameter values resulted. It should be noted that the similarity between images was quite high (Dice coefficient = 0.90), so it is unknown what level of differences in ROI similarity would lead to unacceptable levels of reliability. However, the findings in this investigation highlight the strength of the SFA method in that it is more robust to variances in individual pixel intensities and ROI differences between raters [23].

When accounting for the entire procedure, the test-retest reliability was the most variable across muscles and SFA parameters. However, the majority of parameters had good reliability (ICC(3,1) > 0.80) between trials (Table 3), which is consistent with investigations using tendon [34]. It should be noted that the sonographer for the current study was given no instruction to try and capture the exact same images between trials, but was only instructed to capture images at the same locations. Despite the known variability in image capture with US, the reliability of the SFA method and subsequent extracted parameters was good. The Mmax parameter in the BFlh and SM muscles had the lowest repeatability between trials, which is not surprising. This parameter is a measure of the maximum frequency of the most prominent banded pattern in the image and is therefore the most sensitive to out-of-plane alignment. It is possible that the pennate structure of both BFlh and SM muscles compared to the ST [20,41], could make image acquisition more variable. However, the current test-retest results have similar measures of agreement—especially the Sum parameter (ICC(3,1) > 0.81 for all muscles)—as another study investigating the test-retest reliability (ICC(1,1) = 0.74–0.90) of echo-intensity measures in the hamstrings [42].

To date, this SFA method has been limited in application to patellar or Achilles tendons and one study of the supraspinatus tendon [15,16,34,40,43,44]. Thus, some initial considerations had to be made when adapting SFA to muscle tissue. The first corresponded to the parent ROI selection. In this investigation, each rater was instructed to draw the ROI in the central portion of the image, approximately 0.5 cm from the lateral edges of the images to prevent artefact and avoid resolution drop-off on the lateral sides [37]. Additionally, the superficial and deep boundaries of the ROI were selected approximately 3–4 mm from the superficial and deep aponeuroses [26]. This distance was chosen to capture the tissue that best represented the muscle architecture and minimize changes in muscle architecture as the fascicles tend to curve near the aponeuroses [26,45]. Secondly, previous investigations in tendon have used a kernel size of 32 x 32 pixels corresponding to 2 x 2 mm square. However, the tendons in these investigations had different expected fascicle organization (i.e. different dominant spacing seen in the speckle pattern) and different mean thicknesses ranging from 3–5 mm [15,34]. The thickness of the hamstring muscles range anywhere from 2–4 cm in previous cadaveric dissection [19,20,41] and US studies in young, active individuals [46,47]. After reviewing several training images, a kernel size of 96 x 96 pixels, which corresponded to a square with 6.6 mm sides, was deemed an appropriate size to capture the cumulative image pattern of several fascicles within the kernel. This kernel size is consistent with a previous investigation of frequency analysis of echo-intensity in US images of the quadriceps, which used a single ROI of 64 x 64 pixels corresponding to a square with 6.4 mm sides [9]. However, the SFA method used in the current study differs from this previous study in that spatial frequencies were analyzed in both axial and lateral directions compared to only a single direction [9].

The SFA method leverages information obtained from the frequency domain as it relates to the overall tissue structure. The hierarchical structure of skeletal muscle is well documented, and is comprised of muscle fibers grouped together in fascicles. The anisotropic structure of the muscle due to the fibers and their surrounding perimysium connective tissue, therefore, results in an observed speckle pattern of longer correlation length in the direction of the fascicles compared to the normal direction [14]. This is observed in normal sonographic images as parallel striations of hypoechoic muscle fibers and hyperechoic perimysium [48]. Analysis of the spatial frequency spectrum allows for quantification of the speckle pattern as it relates to the light-dark banding pattern observed in longitudinal B-mode images of healthy muscle.

In this study, we reported four different spatial frequency parameters extracted from the Fourier spectrum (Fig 1, Table 1). To date, most previous studies have investigated the peak spatial frequency radius (PSFR) as a means to classify tendinopathy [15,16,43]. This parameter measures the dominant spacing of the reflected banded pattern. For muscle, PSFR would therefore be expected to indicate the dominant spacing between parallel reflections of muscle fibers and perimysium. Thus, PSFR may prove useful in detecting changes in muscle due to hypertrophy, swelling, localized edema, or mechanical disruption of the permysium [49–51]. The other SFA parameters have not been as widely investigated. The Mmax and Mmax % parameters are complementary in describing the strength of the banded pattern. That is, if Mmax is large, then the banded pattern should be very prominent compared to image background (speckle). Therefore, Mmax and Mmax % might prove useful in detecting deviations from the normal prominence of the striated fascicles in the case of injury or pathology [1,52]. For example, in the case of muscle injury where the perimysium is disrupted, the prominent banding pattern would have an amplitude closer to the background, resulting in a lower Mmax % (i.e. isotropy throughout the imaged tissue). The Sum parameter is most similar to other measures of gray-scale analysis such as echo-intensity, but is the sum total of frequency amplitudes in both axial and lateral directions rather than mean pixel intensity. Although the PSFR has been primarily studied to this point, other parameters described in this investigation may provide added significance and complement PSFR when assessing tissue disruption in the case of tendinopathies or muscle strain injuries.

As this is the first study to use the SFA method in skeletal muscle, the clinical utility of this method in muscle has not yet been fully determined. We believe the current work is an important first step in a potential clinical tool. In order to elucidate possible uses, future studies should investigate any modulation of muscle tissue structure, which impacts functional capacity of the tissue [53]. For example, in the case of hamstring strain injuries, physicians typically assess pain in the posterior thigh and determine if any functional loss is present. Clinical assessments may be corroborated by imaging modalities [54]. In the case of US, the area of maximum tenderness may be imaged and reviewed to determine the presence and extent of any deviation from the normal anatomical structure [52,55,56]. If imaging is repeated following treatment, changes in the size of the injury and echogenicity of the B-mode image will typically be determined. However, changes in muscle architecture will not be quantified. Analysis of B-mode images with SFA may therefore provide objective determination of tissue organization as a result of injury and treatment.

Study limitations should be noted when interpreting the results of this investigation. Previous investigations using SFA have been performed mainly on superficial structures such as the Achilles and patellar tendons [14,15,27,34] or where the depth of the transmit focus point was not reported when assessing the supraspinatus tendon [40]. It is unknown how altering machine settings may influence SFA parameters. Other investigations in quantitative US analyses have highlighted the necessity of calibrated imaging systems for comparison across studies [37,57]. For this reason, US settings were kept constant between all subjects to provide standardized imaging. This may not be feasible in clinical investigations using these methods nor provide the best images between subjects of different body compositions. Future studies should investigate the influence of different machine settings on SFA parameters. Additionally, only young, active individuals were recruited for this study. It is unknown if these measures of reliability also apply to other populations, such as those with pathology or elderly individuals [38].

## Conclusions

Spatial frequency analysis is a quantitative US method which has been previously used to assess intra-tendinous structure. This study has adapted the SFA method and assessed its reliability in the assessment of muscle tissue structure. The intra- and inter-reliability SFA was excellent with good to moderate test-retest agreement. This study indicates that SFA is a reliable method in assessing muscle tissue structure, as characterized by spatial frequency parameters. This method may prove to be useful in future studies in assessing differences in muscle structure following injury, pathology, or throughout rehabilitation.

## Supporting information

S1 Data (XLSX)

## References

[1] Guermazi A, Roemer FW, Robinson P, Tol JL, Regatte RR, Crema MD. Imaging of muscle injuries in sports medicine: Sports imaging series. Radiology. 2017;282: 646–663. 10.1148/radiol.2017160267 28218878

[2] Henderson REA, Walker BF, Young KJ. The accuracy of diagnostic ultrasound imaging for musculoskeletal soft tissue pathology of the extremities: a comprehensive review of the literature. Chiropr Man Therap. 2015;23: 31 10.1186/s12998-015-0076-5 26543553 PMC4634582

[3] van Schie HTM, Bakker EM, Jonker AM, Van Weeren PR. Ultrasonographic tissue characterization of equine superficial digital flexor tendons by means of gray level statistics. Am J Vet Res. 2000;61: 210–219. 10.2460/ajvr.2000.61.210 10685695

[4] van Schie HTM, Bakker EM, Jonker AM, Van Weeren PR. Efficacy of computerized discrimination between structure-related and non-structure-related echoes in ultrasonographic images for the quantitative evaluation of the structural integrity of superficial digital flexor tendons in horses. Am J Vet Res. 2001;62: 1159–1166. 10.2460/ajvr.2001.62.1159 11453496

[5] van Schie HTM, Bakker EM, Jonker AM, van Weeren PR. Computerized ultrasonographic tissue characterization of equine superficial digital flexor tendons by means of stability quantification of echo patterns in contiguous transverse ultrasonographic images. Am J Vet Res. 2003;64: 366–375. 10.2460/ajvr.2003.64.366 12661879

[6] Gao J, Memmott B, Poulson J, Harmon B, Hammond C. Quantitative Ultrasound Imaging to Assess Skeletal Muscles in Adults with Multiple Sclerosis: A Feasibility Study. J Ultrasound Med. 2019;38: 2915–2923. 10.1002/jum.14997 30912176

[7] Santos R, Valamatos MJ, Mil-Homens P, Armada-da-Silva PAS. Muscle thickness and echo-intensity changes of the quadriceps femoris muscle during a strength training program. Radiography. 2018;24: e75–e84. 10.1016/j.radi.2018.03.010 30292517

[8] Pillen S, Tak RO, Zwarts MJ, Lammens MMY, Verrijp KN, Arts IMP, et al Skeletal Muscle Ultrasound: Correlation Between Fibrous Tissue and Echo Intensity. Ultrasound Med Biol. 2009;35: 443–446. 10.1016/j.ultrasmedbio.2008.09.016 19081667

[9] Nishihara K, Kawai H, Hayashi H, Naruse H, Kimura A, Gomi T, et al Frequency analysis of ultrasonic echo intensities of the skeletal muscle in elderly and young individuals. Clin Interv Aging. 2014;9: 1471–8. 10.2147/CIA.S67820 25228800 PMC4160316

[10] Hu CF, Chen CPC, Tsai WC, Hu LL, Hsu CC, Tseng ST, et al Quantification of skeletal muscle fibrosis at different healing stages using sonography. Journal of Ultrasound in Medicine. American Institute of Ultrasound in Medicine; 2012 pp. 43–48. 10.7863/jum.2012.31.1.43 22215768

[11] Fukumoto Y, Ikezoe T, Yamada Y, Tsukagoshi R, Nakamura M, Mori N, et al Skeletal muscle quality assessed from echo intensity is associated with muscle strength of middle-aged and elderly persons. Eur J Appl Physiol. 2012;112: 1519–1525. 10.1007/s00421-011-2099-5 21847576

[12] Torriani M, Kattapuram S V. Musculoskeletal ultrasound: an alternative imaging modality for sports-related injuries. Top Magn Reson Imaging. 2003;14: 103–11. 10.1097/00002142-200302000-00008 12606872

[13] Douis H, Gillett M, James SLJ. Imaging in the diagnosis, prognostication, and management of lower limb muscle injury. Semin Musculoskelet Radiol. 2011;15: 27–41. 10.1055/s-0031-1271957 21332018

[14] Bashford GR, Tomsen N, Arya S, Burnfield JM, Kulig K. Tendinopathy discrimination by use of spatial frequency parameters in ultrasound B-mode images. IEEE Trans Med Imaging. 2008;27: 608–615. 10.1109/TMI.2007.912389 18450534

[15] Kulig K, Landel R, Chang YJ, Hannanvash N, Reischl SF, Song P, et al Patellar tendon morphology in volleyball athletes with and without patellar tendinopathy. Scand J Med Sci Sport. 2013;23: e81–e88. 10.1111/sms.12021 23253169

[16] Kulig K, Chang Y-J, Winiarski S, Bashford GR. Ultrasound-based tendon micromorphology predicts mechanical characteristics of degenerated tendons. Ultrasound Med Biol. 2016;42: 664–673. 10.1016/j.ultrasmedbio.2015.11.013 26718836

[17] R Core Team. R: A language and environment for statistical computing. R Foundation for Statistical Computing Vienna, Austria; 2019.

[18] Zou GY. Sample size formulas for estimating intraclass correlation coefficients with precision and assurance. Stat Med. 2012;31: 3972–3981. 10.1002/sim.5466 22764084

[19] Kellis E, Galanis N, Natsis K, Kapetanos G. Validity of architectural properties of the hamstring muscles: Correlation of ultrasound findings with cadaveric dissection. J Biomech. 2009;42: 2549–2554. 10.1016/j.jbiomech.2009.07.011 19646698

[20] Tosovic D, Muirhead JC, Brown JMM, Woodley SJ. Anatomy of the long head of biceps femoris: An ultrasound study. Clin Anat. 2016;29: 738–745. 10.1002/ca.22718 27012306

[21] Mendes B, Firmino T, Oliveira R, Neto T, Infante J, Vaz JR, et al Hamstring stiffness pattern during contraction in healthy individuals: analysis by ultrasound-based shear wave elastography. Eur J Appl Physiol. 2018;118: 2403–2415. 10.1007/s00421-018-3967-z 30109503

[22] Cepeda CCP, Lodovico A, Fowler N, Rodacki ALF. Effect of an Eight-Week Ballroom Dancing Program on Muscle Architecture in Older Adult Females. J Aging Phys Act. 2015;23: 607–612. 10.1123/japa.2014-0101 25642640

[23] Maurits NM, Bollen AE, Windhausen A, De Jager AEJ, Van Der Hoeven JH. Muscle ultrasound analysis: Normal values and differentiation between myopathies and neuropathies. Ultrasound Med Biol. 2003;29: 215–225. 10.1016/s0301-5629(02)00758-5 12659909

[24] Riggin CN, Sarver JJ, Freedman BR, Thomas SJ, Soslowsky LJ. Analysis of Collagen Organization in Mouse Achilles Tendon Using High-Frequency Ultrasound Imaging. J Biomech Eng. 2013;136: 021029 10.1115/1.4026285 24356929 PMC4023654

[25] Le Sant G, Ates F, Brasseur JL, Nordez A. Elastography study of hamstring behaviors during passive stretching. PLoS One. 2015;10: e0139272 10.1371/journal.pone.0139272 26418862 PMC4587804

[26] Blazevich AJ, Gill ND, Zhou S. Intra- and intermuscular variation in human quadriceps femoris architecture assessed in vivo. J Anat. 2006;209: 289–310. 10.1111/j.1469-7580.2006.00619.x 16928199 PMC2100333

[27] Pearson SJ, Engel AJ, Bashford GR. Changes in tendon spatial frequency parameters with loading. J Biomech. 2017;57: 136–140. 10.1016/j.jbiomech.2017.03.017 28410739

[28] Timmins RG, Shield AJ, Williams MD, Lorenzen C, Opar DA. Biceps femoris long head architecture: A reliability and retrospective injury study. Med Sci Sports Exerc. 2015;47: 905–913. 10.1249/MSS.0000000000000507 25207929

[29] Timmins RG, Bourne MN, Shield AJ, Williams MD, Lorenzen C, Opar DA. Short biceps femoris fascicles and eccentric knee flexor weakness increase the risk of hamstring injury in elite football (soccer): A prospective cohort study. Br J Sports Med. 2016;50: 1524–1535. 10.1136/bjsports-2015-095362 26675089

[30] Freitas SR, Marmeleira J, Valamatos MJ, Blazevich A, Mil-Homens P. Ultrasonographic Measurement of the Biceps Femoris Long-Head Muscle Architecture. J Ultrasound Med. 2018;37: 977–986. 10.1002/jum.14436 29027683

[31] Zou KH, Warfield SK, Bharatha A, Tempany CMC, Kaus MR, Haker SJ, et al Statistical Validation of Image Segmentation Quality Based on a Spatial Overlap Index. Acad Radiol. 2004;11: 178–189. 10.1016/s1076-6332(03)00671-8 14974593 PMC1415224

[32] Stratford PW, Goldsmith CH. Use of the standard error as a reliability index of interest: An applied example using elbow flexor strength data. Phys Ther. 1997;77: 745–750. 10.1093/ptj/77.7.745 9225846

[33] Weir JP. Quantifying test-retest reliability using the intraclass correlation coefficient and the SEM. J Strength Cond Res. 2005;19: 231–40. 10.1519/15184.1 15705040

[34] Cassel M, Risch L, Mayer F, Kaplick H, Engel A, Kulig K, et al Achilles tendon morphology assessed using image based spatial frequency analysis is altered among healthy elite adolescent athletes compared to recreationally active controls. J Sci Med Sport. 2019;22: 882–886. 10.1016/j.jsams.2019.03.011 31000456

[35] Koo TK, Li MY. A Guideline of Selecting and Reporting Intraclass Correlation Coefficients for Reliability Research. J Chiropr Med. 2016;15: 155–163. 10.1016/j.jcm.2016.02.012 27330520 PMC4913118

[36] Erratum to “A Guideline of Selecting and Reporting Intraclass Correlation Coefficients for Reliability Research” [J Chiropr Med 2016;15(2):155–163]. J Chiropr Med. 2017;16: 346. 10.1016/j.jcm.2016.02.012 27330520 PMC4913118

[37] Zaidman CM, Holland MR, Anderson CC, Pestronk A. Calibrated quantitative ultrasound imaging of skeletal muscle using backscatter analysis. Muscle and Nerve. 2008;38: 893–898. 10.1002/mus.21052 18563722

[38] Strasser EM, Draskovits T, Praschak M, Quittan M, Graf A. Association between ultrasound measurements of muscle thickness, pennation angle, echogenicity and skeletal muscle strength in the elderly. Age. 2013;35: 2377–2388. 10.1007/s11357-013-9517-z 23456136 PMC3824993

[39] Ho K-Y, Baquet A, Chang Y-J, Chien L-C, Harty M, Bashford G, et al Factors related to intra-tendinous morphology of Achilles tendon in runners. PLoS One. 2019;14: e0221183 10.1371/journal.pone.0221183 31412086 PMC6693759

[40] Pozzi F, Seitz AL, Plummer HA, Chow K, Bashford GR, Michener LA. Supraspinatus tendon micromorphology in individuals with subacromial pain syndrome. J Hand Ther. 2017;30: 214–220. 10.1016/j.jht.2017.04.001 28502699

[41] Kellis E, Galanis N, Natsis K, Kapetanos G. Muscle architecture variations along the human semitendinosus and biceps femoris (long head) length. J Electromyogr Kinesiol. 2010;20: 1237–1243. 10.1016/j.jelekin.2010.07.012 20727788

[42] Ruas C V., Pinto RS, Lima CD, Costa PB, Brown LE. Test-Retest Reliability of Muscle Thickness, Echo-Intensity and Cross Sectional Area of Quadriceps and Hamstrings Muscle Groups Using B-mode Ultrasound. Int J Kinesiol Sport Sci. 2017;5: 35–41. 10.7575/aiac.ijkss.v.5n.1p.35

[43] Kulig K, Oki KC, Chang Y-J, Bashford GR. Achilles and patellar tendon morphology in dancers with and without tendon pain. Med Probl Perform Art. 2014;29: 221–228. 10.21091/mppa.2014.4044 25433259

[44] Mersmann F, Pentidis N, Tsai M-S, Schroll A, Arampatzis A. Patellar Tendon Strain Associates to Tendon Structural Abnormalities in Adolescent Athletes. Front Physiol. 2019;10: 963 10.3389/fphys.2019.00963 31427983 PMC6687848

[45] Van Leeuwen JL, Spoor CW. Modelling mechanically stable muscle architectures. Philos Tansactions R Soc London Ser B, Biol Sci. 1992;336: 275–292. 10.1098/rstb.1992.0061 1353268

[46] e Lima KMM, Carneiro SP, de S. Alves D, Peixinho CC, de Oliveira LF. Assessment of Muscle Architecture of the Biceps Femoris and Vastus Lateralis by Ultrasound After a Chronic Stretching Program. Clin J Sport Med. 2015;25: 55–60. 10.1097/JSM.0000000000000069 24451696

[47] Pimenta R, Blazevich AJ, Freitas SR. Biceps femoris long-head architecture assessed using different sonographic techniques. Med Sci Sports Exerc. 2018;50: 2584–2594. 10.1249/MSS.0000000000001731 30067589

[48] Purohit NB, King LJ. Ultrasound of lower limb sports injuries. Ultrasound. SAGE Publications Ltd; 2015 pp. 149–157. 10.1177/1742271X15588809 PMC4760585 27433251

[49] Schoenfeld BJ. The mechanisms of muscle hypertrophy and their application to resistance training. Journal of Strength and Conditioning Research. 2010 pp. 2857–2872. 10.1519/JSC.0b013e3181e840f3 20847704

[50] Peetrons P. Ultrasound of muscles. Eur Radiol. 2002;12: 35–43. 10.1007/s00330-001-1164-6 11868072

[51] Lee JC, Mitchell AWM, Healy JC. Imaging of muscle injury in the elite athlete. British Journal of Radiology. 2012 pp. 1173–1185. 10.1259/bjr/84622172 22496067 PMC3495577

[52] Woodhouse JB, McNally EG. Ultrasound of Skeletal Muscle Injury: An Update. Semin Ultrasound, CT MRI. 2011;32: 91–100. 10.1053/j.sult.2010.12.002 21414545

[53] Lieber RL, Fridén J. Functional and clinical significance of skeletal muscle architecture. Muscle Nerve. 2000;23: 1647–1666. 10.1002/1097-4598(200011)23:11<1647::aid-mus1>3.0.co;2-m 11054744

[54] Kujala UM, Orava S, Järvinen M. Hamstring injuries. Current trends in treatment and prevention. Sports Med. 1997;23: 397–404. 10.2165/00007256-199723060-00005 9219322

[55] Slavotinek JP. Muscle injury: The role of imaging in prognostic assignment and monitoring of muscle repair. Semin Musculoskelet Radiol. 2010;14: 194–200. 10.1055/s-0030-1253160 20486027

[56] Crema MD, Yamada AF, Guermazi A, Roemer FW, Skaf AY. Imaging techniques for muscle injury in sports medicine and clinical relevance. Curr Rev Musculoskelet Med. 2015;8: 154–161. 10.1007/s12178-015-9260-4 25708212 PMC4596169

[57] Pillen S, Van Dijk JP, Weijers G, Raijmann W, De Korte CL, Zwarts MJ. Quantitative gray-scale analysis in skeletal muscle ultrasound: A comparison study of two ultrasound devices. Muscle and Nerve. 2009;39: 781–786. 10.1002/mus.21285 19301363

